# Tobacco Transcription Factor NtWRKY70b Facilitates Leaf Senescence via Inducing ROS Accumulation and Impairing Hydrogen Sulfide Biosynthesis

**DOI:** 10.3390/ijms25073686

**Published:** 2024-03-26

**Authors:** Xinshuang Zhang, Yan Sun, Hao Wu, Ying Zhu, Xin Liu, Songchong Lu

**Affiliations:** College of Life Sciences, Qingdao Agricultural University, Qingdao 266109, China

**Keywords:** leaf senescence, ROS, H_2_S, WRKY, tobacco

## Abstract

Leaf senescence is the terminal stage of leaf development, and its initiation and progression are closely controlled by the integration of a myriad of endogenous signals and environmental stimuli. It has been documented that WRKY transcription factors (TFs) play essential roles in regulating leaf senescence, yet the molecular mechanism of WRKY-mediated leaf senescence still lacks detailed elucidation in crop plants. In this study, we cloned and identified a tobacco WRKY TF gene, designated *NtWRKY70b*, acting as a positive regulator of natural leaf senescence. The expression profile analysis showed that *NtWRKY70b* transcript levels were induced by aging and hydrogen peroxide (H_2_O_2_) and downregulated upon hydrogen sulfide (H_2_S) treatment. The physiological and biochemical assays revealed that overexpression of *NtWRKY70b* (OE) clearly promoted leaf senescence, triggering increased levels of reactive oxygen species (ROS) and decreased H_2_S content, while disruption of *NtWRKY70b* by chimeric repressor silencing technology (SRDX) significantly delayed the onset of leaf senescence, leading to a decreased accumulation of ROS and elevated concentration of H_2_S. The quantitative real-time PCR analysis showed that the expression levels of various senescence-associated genes and ROS biosynthesis-related genes (*NtRbohD* and *NtRbohE*) were upregulated in OE lines, while the expression of H_2_S biosynthesis-related genes (*NtDCD* and *NtCYSC1*) were inhibited in OE lines. Furthermore, the Yeast one-hybrid analysis (Y1H) and dual luciferase assays showed that NtWRKY70b could directly upregulate the expression of an ROS biosynthesis-related gene (*NtRbohD*) and a chlorophyll degradation-related gene (*NtPPH*) by binding to their promoter sequences. Accordingly, these results indicated that NtWYKY70b directly activated the transcript levels of *NtRbohD* and *NtPPH* and repressed the expression of *NtDCD* and *NtCYCS1*, thereby promoting ROS accumulation and impairing the endogenous H_2_S production, and subsequently accelerating leaf aging. These observations improve our knowledge of the regulatory mechanisms of WRKY TFs controlling leaf senescence and provide a novel method for ensuring high agricultural crop productivity via genetic manipulation of leaf senescence in crops.

## 1. Introduction

In plants, leaf senescence is a crucial stage of plant development, which is stimulated by integrating multiple internal factors and external environmental cues [1,2]. During this process, plants have evolved highly elaborate and sophisticated senescence-regulating mechanisms consisting of the fine-tuned modulation of the integration of multiple phytohormones, including abscisic acid (ABA), jasmonic acid (JA), and salicylic acid (SA), and the degradation of subcellular organelles (e.g., chloroplast and mitochondria) and metabolic changes like the hydrolysis of chlorophyll and other macromolecules, e.g., proteins, lipids, and nucleic acids, followed by nutrient remobilization and reallocation throughout the plant’s life cycle to ensure the reproductive success [3,4,5,6]. It is well known that the recycling of nutrient from senescing leaves to new organs, including growing leaves and fruits and developing seed, is a crucial process for plant fitness and crop productivity under fluctuating environmental conditions. An increasing number of reports have demonstrated that precocious leaf senescence negatively influences the yield and quality of crops, while prolongation of leaf longevity remarkably improves the plant biomass and crop yields [2,4,5,6,7,8]. Thus, the proper timing of the initiation and progress of leaf senescence is critical for plant development and crops’ productivity, yet the understanding of how plants sense and respond to internal aging signals and external environmental cues and then initiate leaf senescence is still fragmentary.

Emerging evidence has demonstrated that during leaf senescence, an increasing number of transcription factor (TF) genes from multiple families, such as NAC and WRKY, are significantly upregulated [9,10,11], implying that TFs play essential roles in transcriptional regulation of senescence-associated genes (SAG) in this process. WRKY transcription factors (TFs), defined by the WRKY domain consisting of the conserved amino acid sequence WRKYGQK at its N-terminal, comprise a superfamily of regulatory biomolecules in plants which widely participate in a myriad of key biological processes, including multiple developmental and physiological processes and various biotic and abiotic stress responses [12,13,14,15,16,17]. In Arabidopsis, a transcriptome analysis revealed that numerous WRKY genes were dramatically induced in senescing leaves [11], indicating important roles of WRKY TFs in leaf senescence regulation. Recent genetic and molecular biological investigation have provided various evidence to demonstrate that WRKY members play vital roles in the coordinated regulation of leaf senescence. For example, overexpression of several WRKY genes such as AtWRKY75, AtWRKY45, AtWRKY55, and AtWRKY42 positively influences leaf senescence, while the disruption of these TFs genes prolongs leaf longevity [9,18,19,20,21]. Conversely, AtWRKY25 and AtWRKY70 act as negative regulators of modulation of leaf senescence [22,23,24]. Recently, WRKY47 and WRKY70, from Brassica napus, were identified as participating in controlling leaf senescence [17,25].

Studies concerning free radical damage reactions in plant have revealed that reactive oxygen species (ROS) have diverse functions in various developmental and physiological processes, including seed germination, root morphogenesis, stomatal movement, leaf development, and abiotic stress responses [4,18,26,27,28]. It has been reported that respiratory burst oxidase homologs (Rbohs), as homologs of mammalian NADPH oxidases, participate in accumulation of apoplastic ROS (e.g., O_2_^−^ and H_2_O_2_), which are targeted to the plasma membrane [29]. In Arabidopsis, *Rbohs* genes class consists of 10 members, namely *AtRbohA*–*AtRbohJ* [29]. Recent studies have demonstrated that multiple *Rbohs* genes (e.g., *RbohD, RbohE,* and *RbohF*) are involved in various physiological processes and biotic/abiotic stress responses in Arabidopsis, tobacco, rice, and oilseed rape [30,31,32,33,34,35]. Numerous studies have reported that high levels of endogenous ROS are a typical physiological feature in aging leaves, and their burst leads to a disturbance of redox state and dramatic oxidative damage to the cell membrane, ultimately causing leaf senescence and cell death in plants [32,36,37]. Consequently, ROS play a vital function in regulating the onset and progress of leaf aging, yet little is known about the upstream transcriptional network which is involved in controlling the accumulation of ROS during leaf senescence.

Hydrogen sulfide (H_2_S) is a colorless, highly soluble gas with a foul odor similar to rotten eggs, which has been regarded as a toxic gas for many years [38]. Recently, in plants, it has been understood that H_2_S, as the third key endogenous gasotransmitter besides nitric oxide (NO) and carbon monoxide (CO), has important functions in a myriad of developmental and physiological processes, including root development, seedlings growth, stomatal movement, flowering time, leaf senescence, fruit ripening, and plant responses to abiotic stresses (e.g., drought, salt, low temperature, and osmotic stresses) [27,39,40,41,42,43,44,45,46,47,48]. Furthermore, emerging studies have demonstrated that H_2_S influences various signaling pathways via increased levels of the persulfidation of its target proteins. For example, in Arabidopsis, H_2_S was reported to modulate the ABA signaling network by improving the persulfidation of SnRK2.6 and ABI4, two curial components of the ABA signal transduction, leading to these proteins’ functions changing [43,44]. In heading Chinese cabbage, H_2_S was found to be involved in controlling flowering by s-sulfhydration of BraFLCs, some key MADs box transcription factors for regulating the timing of flowering, thereby changing their binding ability to downstream promoters, subsequently causing early flowering [45].

Genetic and molecular studies have revealed that WRKY TFs, ROS, and H_2_S have vital functions in the modulation of leaf senescence [26,46,49]. However, the molecular mechanism driving their relationships in the leaf aging process remains largely unknown. Tobacco is an important economic crop and also a significant model organism for life science research, yet little is understood on the molecular mechanisms of leaf senescence in tobacco. Based our previous transcriptome assay, in this study, we isolated and identified a WRKY TF gene, namely NtWRKY70b, which positively influenced leaf senescence through modified levels of ROS and H_2_S in tobacco. Our investigations revealed that NtWRKY70b acts as a new positive regulator in regulating leaf senescence through modulating the metabolism of ROS and H_2_S.

## 2. Results

### 2.1. Identification and Sequence Analysis of NtWYKY70b Orthologs

**Figure 1:**
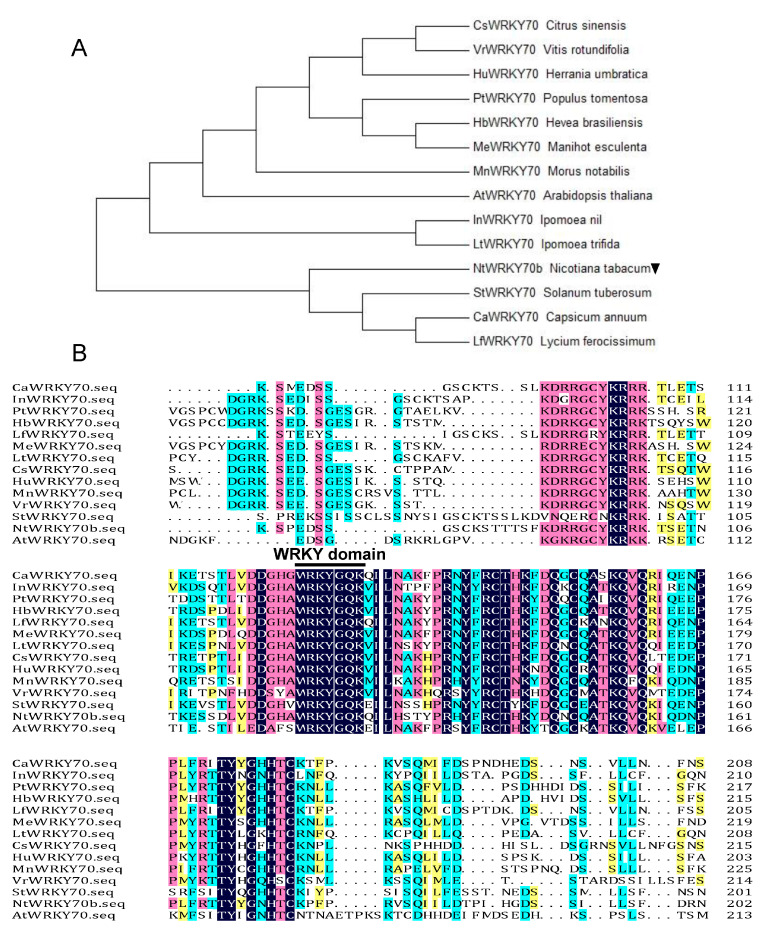
Bioinformatics analysis and characterization of NtWRKY70b. (**A**) Phylogenetic analysis of WRKY70 orthologs from different species of plants utilizing the neighbor-joining (NJ) method in MEGA 7.0. NtWRKY70b is marked by a downward black triangle. (**B**) Sequences alignment of NtWRKY70b with 13 other WRKY70-like homologous protein sequences. The conserved core motif WRKYGQK in the WRKY domain is indicated by a black line. The NCBI accession numbers of these different WRKY homologous proteins are listed in Supplementary Table S1.

Based on our previous transcriptome data designed to screen senescence-associated genes with senescent leaves from the Nicotiana tabacum “K326” [6], we isolated a senescence-induced transcription factor gene named *NtWYKY70b*. According to the data of the National Center for Biotechnology Information (NCBI) and the Solanaceae Genomics Network, we searched the homologous genes of *NtWRKY* in other species and performed a multiple alignment and phylogenetic evolution analysis of these sequences by the DNAMAN and Neighbor-Joining (NJ) method, respectively. The results showed that NtWRKY70b encoded a 300-amino-acid protein containing conserved WRKY domains, including the WRKYGQ/KK core motif (Figure 1A,B), whose amino acid sequence was shown to be highly homologous to AtWRKY70 and LfWRKY70. Thus, the NtWRKY70b could be a senescence-associated WRKY transcription factor.

### 2.2. Expression of NtWRKY70b Is Upregulated during Leaf Senescence

**Figure 2:**
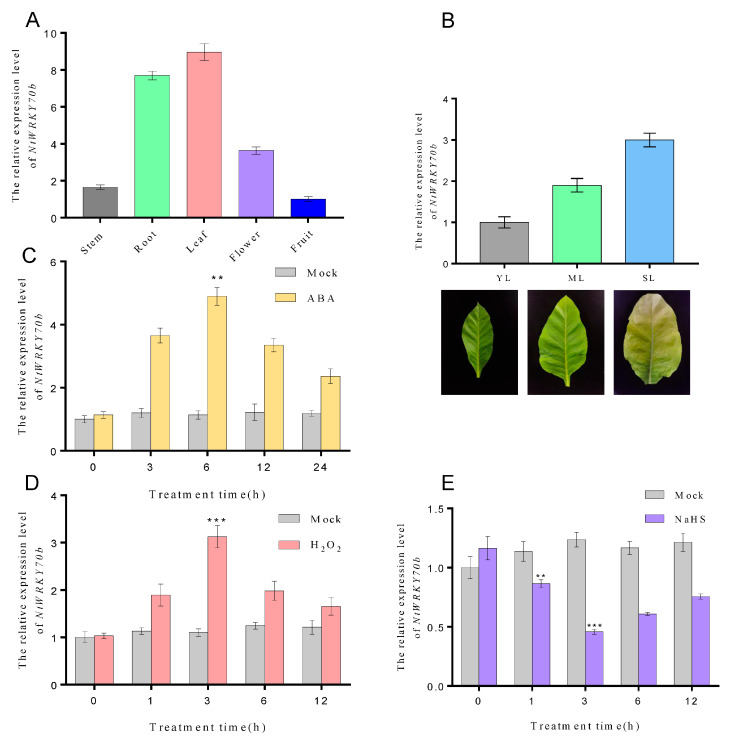
Transcriptional patterns of *NtWRKY70b* in tobacco. (**A**,**B**) The expression levels of NtWRKY70b in various tissues of tobacco “K326”, such as stem, root, leaf, flower, and fruit (**A**), and in leaves at different stages of development, including young leaves YL, mature leaves ML, and senescent leaves SL (**B**). (**C**–**E**) The expression patterns of NtWRKY70b in 12-day-old “K326” seedlings under exogenous molecules treatments: ABA (100 μM), NaHS (10 μM), H_2_O_2_ (10 mM), and water as control (Mock). The values are represented as means ± SD, *n* = 3. Asterisks indicate statistically significant differences levels (Student’s *t* test, ** *p* < 0.01, and *** *p* < 0.001) with corresponding controls.

Our previous transcriptome data showed that the expression levels of *NtWRKY70b* were significantly elevated during natural aging. In order to deeply explore the spatiotemporal expression profiles of *NtWRKY70b*, we performed quantitative real-time PCR (qRT-PCR) to detect its transcript level in various tissues (e.g., root, stem, flower, fruit, young leaf, mature leaf, and senescent leaf) and under multiple abiotic stress conditions. As the results indicate, *NtWRKY70b* was expressed ubiquitously in all tissues mentioned above, and its expression was highest in the leaf, followed by root, flower, and stem, with the lowest level in fruit (Figure 2A). Furthermore, we found that the transcript level of *NtWRKY70b* was gradually increased during leaf aging processes (Figure 2B). Under ABA and H_2_O_2_ conditions, the expression of *NtWRKY70b* was strongly upregulated. As the qRT-PCR analysis showed, the expression levels of NtWRKY70b began to accumulate after 3 h of exposure to ABA treatment and peaked after 6 h of ABA treatment, exhibiting a five-fold increase compared to that in control check (CK) plants, and then declined gradually. Similarly, when plants were exposed to H_2_O_2_ treatment, the transcript levels of NtWRKY70b were significantly induced compared to that in CK plants (Figure 2C,D). Conversely, H_2_S treatment caused a decreased expression level of NtWRKY70b. Upon application of exogenous sodium hydrosulfide (NaHS, a H_2_S donor), the expression levels of *NtWRKY70b* started to decline after 1 h of NaHS treatment, and bottomed out after 3 h of exposure to NaHS, showing a 0.4-fold decrease relative to that of the CK group (Figure 2E). In summary, these findings indicated that *NtWRKY70b* might be involved in regulating leaf senescence and the abiotic stress response in plants through multiple signaling networks (e.g., ABA, ROS, and H_2_S).

### 2.3. NtWRKY70b Is Localized in the Nucleus and Acts as a Transcriptional Activator

**Figure 3:**
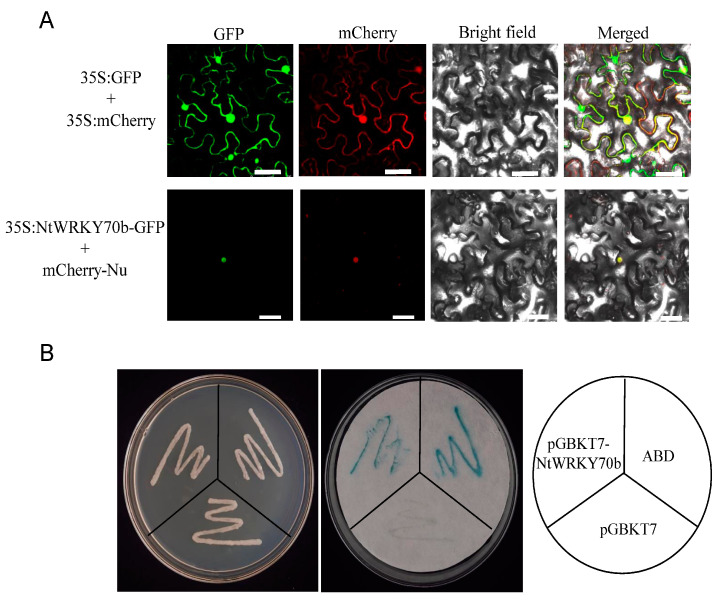
Transcription factor characterization of NtWRKY70b. (**A**) NtWRKY70b is targeted in the nucleus in cells. *NtWRKY70b-GFP, Empty GFP*, and a nuclear marker (*BES1n-mCherry*) were expressed in the leaves of *N. benthamiana* (bars for 50 µm). (**B**) Transcriptional activation assay of NtWRKY70b in the yeast AH109. protein. The pGBKT7-AD performed the role of the positive control, while empty pGBKT7 was the negative control. The plates were incubated for 3 days and then subjected to the galactosidase assay.

To investigate the subcellular localization of NtWRKY70b protein, we performed a green fluorescent protein (GFP)-labeled assay for the study of subcellular localization. The *35S:NtWRKY70b* was transformed into leaves of *N. benthamiana* by the agrobacterium-mediated method. The fluorescent observation data showed that the NtWRKY70b-GFP signal was positioned in the nucleus, which was co-localized with the known nuclear marker BES1n-mCherry (Figure 3A), while the empty GFP was ubiquitously distributed throughout the cells. These results indicated that the NtWRKY70b was directly targeted to the nucleus of cells.

To further test whether NtWRKY70b has the ability to activate or repress transcription, we constructed a recombinant yeast expression cassette *pGBKT7-NtWRKY70b*, then transformed it into the AHA109 yeast strain. As our observations show, all yeast transformants could grow normally on the plate containing SD/-Trp, which showed that these vectors had been successfully transformed into yeast cells. As a positive result of the transactivation assay, β-galactosidase gene (*lacZ*) is expressed. Yeast colonies that express *lacZ* turn blue in the presence of the chromogenic substrate X-β-Gal. The galactosidase assays showed that these yeast colonies carrying *pGBKT7-NtWRKY70b* and *pGBKT7-AD* displayed a remarkable blue color, while the empty control colonies did not appear blue (Figure 3B). The experiment confirmed that NtWRKY70b acted as a transcriptional activator to induce the expression of the β-gal reporter gene. Thus, the above results suggested that NtWRKY70b is a nuclear-localized transcriptional activator in cells.

### 2.4. NtWRKY70b Accelerates Dark-Induced Leaf Senescence

**Figure 4:**
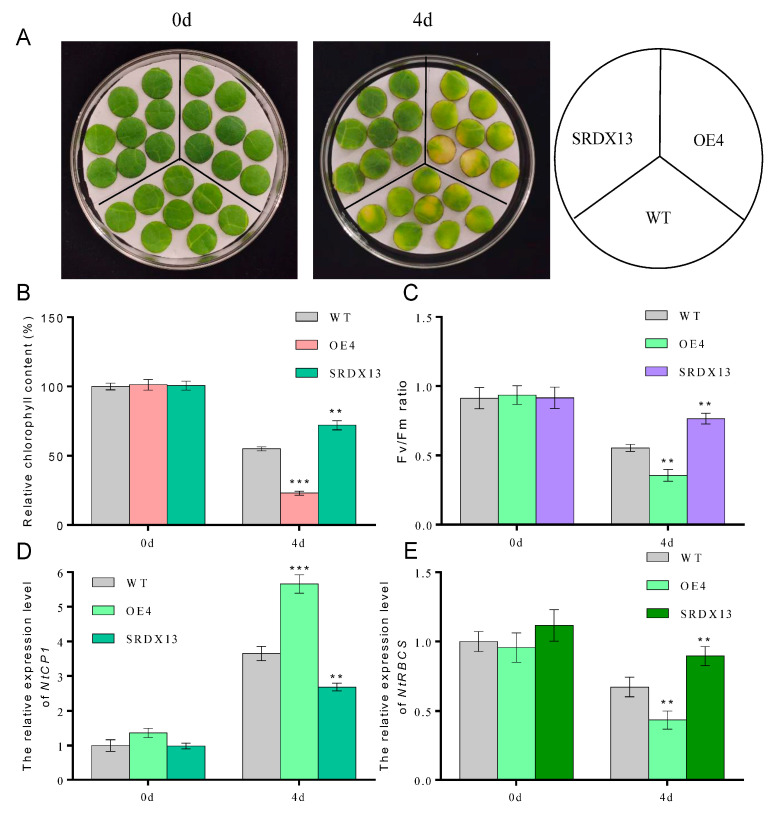
Overexpression of *NtWRKY70b* accelerates dark-induced leaf senescence. (**A**) Phenotype observations of ten-week-old and 8th leaves of *NtWRKY70b-OE, NtWRKY70b-SRDX*, and WT tobacco plants “K326” for four days under dark conditions. (**B**,**C**) Chlorophyll content (**A**) and Fv/Fm (**C**) of leaves in (**A**). (**D**,**E**) The expression level of *NtCP1* (**D**) and *NtRBCS* (**E**) of these transgenic lines in (**A**). The values are represented as means ± SD, n = 3. Asterisks indicate statistically significant differences levels (Student’s *t* test, ** *p* < 0.01, and *** *p* < 0.001) with corresponding controls.

It is well known that dark treatment is often used to effectively accelerate the induction of leaf senescence [4,6]. To elucidate the potential biological roles of NtWRKY70b in senescence processes, we constructed overexpression cassettes *35S:NtWRKY70b* (OE) and the dominant-negative vectors *35S:NtWRKY70b-SRDX* (SRDX) and then transiently transformed these vectors into the tobacco (N. *benthamiana*) leaves. The qRT-PCR analysis showed that the transcript level of *NtWRKY70b* in transgenic leaves was increased approximately 23-fold relative to its level in the leaves containing the empty vector (CK). After the above-mentioned transformed tobacco leaves were subjected to dark treatment for 3 days, it was found that OE leaves showed an early yellowing phenomenon compared to CK (Supplementary Figure S1A,B). Moreover, the relative chlorophyll content and Fv/Fm in OE leaves were dramatically decreased compared with that in CK, and overexpression of *NtWRKY70b* triggered an elevated level of relative electrolytic leakage. However, the SRDX leaves showed the opposite phenotype (Supplementary Figure S1C–E). In addition, we obtained independent *NtWRKY70b*-overexpressing plants (12 lines) and *NtWRKY70b:SRDX* plants (10 lines) based on common tobacco “K326” by the agrobacterium-mediated method. To further test the key function of NtWRKY70b in darkness-induced leaf senescence, two independent transgenic lines (*OE4* and *SRDX13)* were chosen to undergo a similar darkness treatment. In parallel with the findings in those transiently transgenic leaves, after exposure to darkness conditions for 4 days, transgenic line *OE4* displayed significantly accelerated leaf aging relative to wild-type plants (WT), while *SRDX13* plants regulated leaf senescence negatively. Moreover, the physiological analysis of chlorophyll content and Fv/Fm ratio in those transgenic plants demonstrated that NtWRKY70b plays positive roles in the regulation of dark-triggered senescence (Figure 4A–C). We also detected the transcript levels of *CYSTEINE PROTEINASE 1* (*NtCP1*, *SENESCENCE-ASSOCIATED GENE 12/SAG12* homolog in tobacco) and the *RIBULOSE BISPHOSPHATE CARBOXYLASE SMALL CHAIN* (*NtRBCS*), two well-known senescence-associated genes [6]. The expression of *NtCP1* was remarkably increased in the overexpressing lines *OE4* and decreased in the *SRDX13* lines compared with that in WT, while the expression trend of *NtRBCS* in *OE4* and *SRDX13* lines was opposite to that of *NtCP1* (Figure 4D,E). Therefore, these results indicate that NtWRKY70b takes part in promoting dark-induced leaf senescence.

### 2.5. NtWRKY70b Promotes Age-Triggered Leaf Senescence via Modulating Levels of ROS and H_2_S

**Figure 5:**
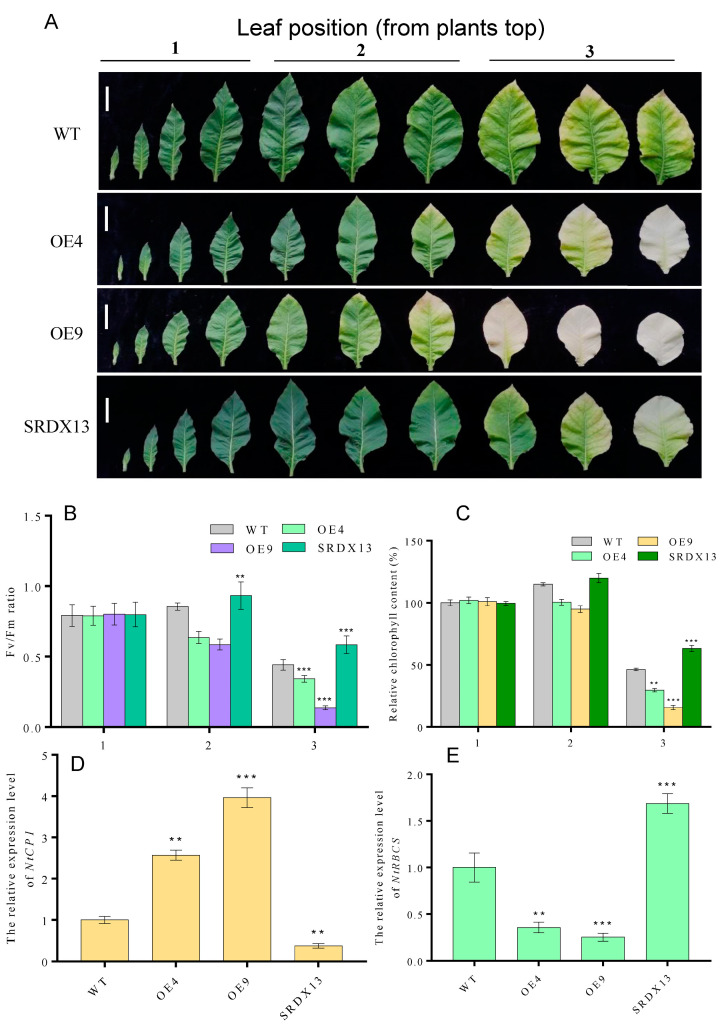
Overexpression of *NtWRKY70b* promotes age-dependent leaf senescence. (**A**) Phenotype of twelve-week-old transgenic plants ((bars for 5 cm)). (**B**,**C**) Chlorophyll content (**B**) and Fv/Fm (**C**) of leaves in (**A**). (**D**,**E**) The expression level of *NtCP1* (**D**) and *NtRBCS* (**E**) of these transgenic lines in (**A**). (1) The first to fourth leaves as counted from the top, (2) the fifth to seventh leaves from the top, and (3) the eighth to tenth leaves from the top. The values are represented as means ± SD, *n* = 3. Asterisks indicate statistically significant differences levels (Student’s *t*-test, ** *p* < 0.01, and *** *p* < 0.001) with corresponding controls.

**Figure 6:**
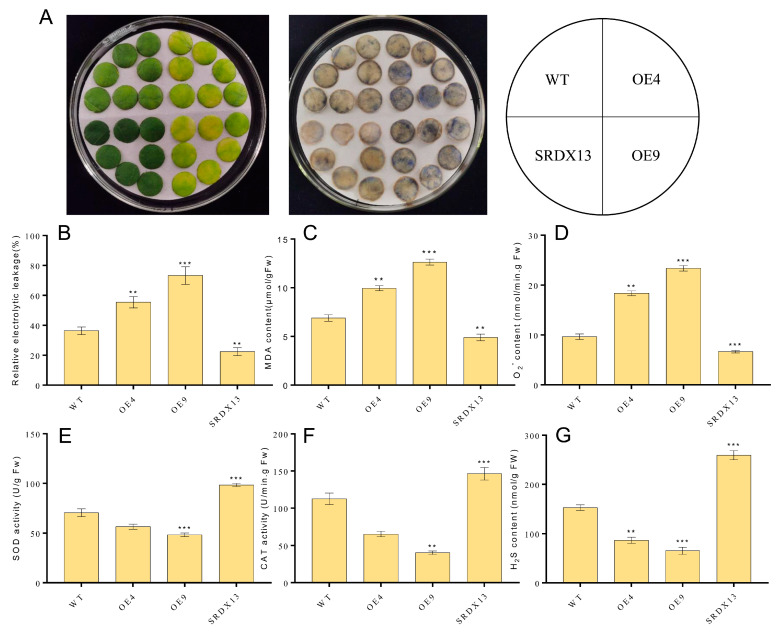
NtWRKY70b regulates ROS and H_2_S metabolism during leaf senescence. (**A**) NBT staining. Three independent experiments were conducted (n ≥ 20), showing similar results. (**B**) Ions leakage rate. (**C**) MDA content. (**D**) O_2_^.−^ content. (**E**,**F**) Activity analysis of antioxidant enzymes SOD € and CAT (**F**) in transgenic plants and WT. (**G**) H_2_S content. The data are means ± SD of three biological replicates. Asterisks indicate statistically significant differences levels (Student’s *t* test, ** *p* < 0.01, and *** *p* < 0.001) with corresponding controls.

As described above, the expression of *NtWRKY70b* was upregulated gradually during natural senescence. In order to further explore whether *NtWRKY70b* is involved in regulating the progress of leaf aging, we first studied the aging phenotype of *NtWRKY70b* transgenic lines under normal growth conditions. The results showed that compared with WT, the *NtWRKY70b*-overexpressing lines *OE4* and *OE9* showed an obviously premature senescence phenotype, while the disruption of *NtWRKY70b* function caused delayed leaf senescence (Figure 5A). In order to further verify the above aging phenotype from the physiological level, we tested the relative chlorophyll content and Fv/Fm ratio of these transgenic lines. The results showed that the relative chlorophyll content and Fv/Fm ratio of *OE4* and *OE9* were significantly lower than those of WT, while the relative chlorophyll content and Fv/Fm ratio of *SRDX13* plants were increased significantly compared with that of WT (Figure 5B,C). Furthermore, the expression of senescence marker genes *NtCP1* and *NtRBCS* in transgenic lines is shown in Figure 5D,E. In *OE4* and *OE9* lines, the expression of *NtCP1* is higher, while the expression of *NtRBCS* is lower, compared to WT. Numerous reports have demonstrated that excess ROS induces leaf senescence. To determine whether NtWRKY70b influences ROS accumulation in the leaf senescence process, we detected the ROS metabolism by NBT staining, relative electrolytic leakage, malondialdehyde (MDA), and activity analysis of multiple antioxidases. The observation indicated that overexpressing lines *OE4* and *OE9* contained a higher accumulation of O_2_^−^ than WT, while *SRDX13* had a lower level of ROS relative to WT. Moreover, the relative electrolytic leakage and malondialdehyde (MDA) were significantly higher in *OE4* and *OE9* than those in WT (Figure 6A–D). We also tested the activities of some antioxidases, including superoxide dismutase (SOD) and catalase (CAT) (Figure 6E,F). There were decreases in the activities of SOD and CAT in the *OE4* and *OE9* lines, leading to elevated levels of ROS. According to the NaHS-reduced expression of *NtWRKY70b* mentioned above, we detected endogenous H_2_S production in *OE*, *SRDX*, and WT plants during leaf senescence. As shown in Figure 6G, the H_2_S content in *NtWRKY70b-OE* lines showed a significant decrease compared to that in WT, while there was more H_2_S production in *NtWRKY70b-SRDX* lines. It can be postulated that NtWRKY70b plays essential functions in promoting leaf senescence via regulating the accumulation of ROS and H_2_S scavenging.

### 2.6. NtWRKY70b Regulates the Expression of Synthesis-Related Genes of ROS and H_2_S

**Figure 7:**
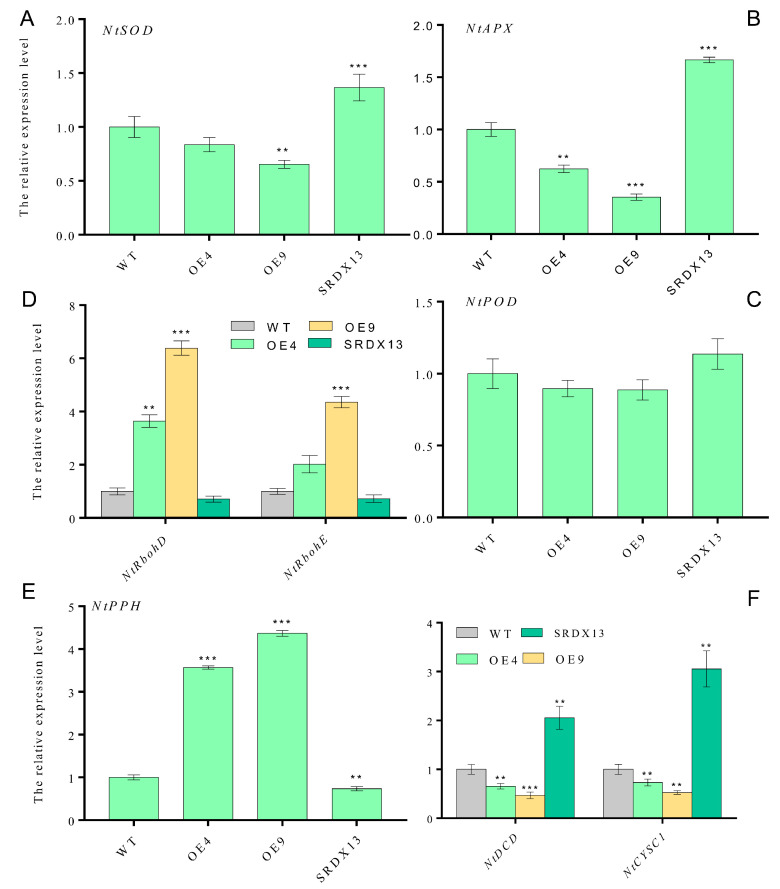
NtWRKY70b influences the expression levels of some biosynthesis genes of ROS and H_2_S. (**A**) *NtSOD*. (**B**) *NtAPX*. (**C**) *NtPOD*. (**D**) *NtRbohD and NtRbohE*. (**E**) *NtPPH*. (**F**) *NtDCD* and *NtCYSC1*. The 8th leaves were harvested from 12-week-old transgenic plants grown under natural long-day conditions in soil and were used for qRT-PCR analysis. The expression levels of these genes were normalized to that of actin, an internal control. The data are means ± SD of three biological replicates. Asterisks indicate statistically significant differences levels (Student’s *t* test, ** *p* < 0.01, and *** *p* < 0.001) with corresponding controls.

To further decipher the molecular mechanism of NtWRKY70b influencing leaf senescence, through qRT-PCR analysis, we determined the transcriptional level of some ROS-synthesis/scavenging-related genes (e.g., *NtRbohD*, *NtRbohE*, *NtSOD*, *NtAPX*, and *NtPOD*), H_2_S-synthesis-related genes (e.g., *NtDCD* and *NtCYSC1*), and chlorophyll-catabolic genes (e.g., *NtPPH*). These results showed that the expression levels of ROS-scavenging-related genes (*NtSOD*, *NtAPX*, and *NtPOD*) were decreased in overexpressing plants (OE4 and OE9) compared with that in WT (Figure 7A–C), but there was an obvious increase in *NtRbohD* and *NtRbohE* transcription in overexpressing plants (Figure 7D), thereby triggering excess ROS production. In addition, we found that the expression level of *NtDCD1* and *NtCYSC1*, two novel H_2_S biosynthesis genes, were significantly downregulated in *NtWRKY70b-OE* plants (Figure 7F), implying that NtWRKY70b inhibited the production of H_2_S through directly or indirectly regulating the expression of *NtDCD1* and *NtCYSC1*. In summary, these results demonstrates that NtWRKY70b regulates the accumulation of ROS and H_2_S via directly or indirectly regulating the expression of their synthesis-related genes, subsequently promoting natural leaf senescence.

### 2.7. NtWRKY70b Directly Regulates Expression of NtRbohD and NtPPH by Binding to Their Promoters

**Figure 8:**
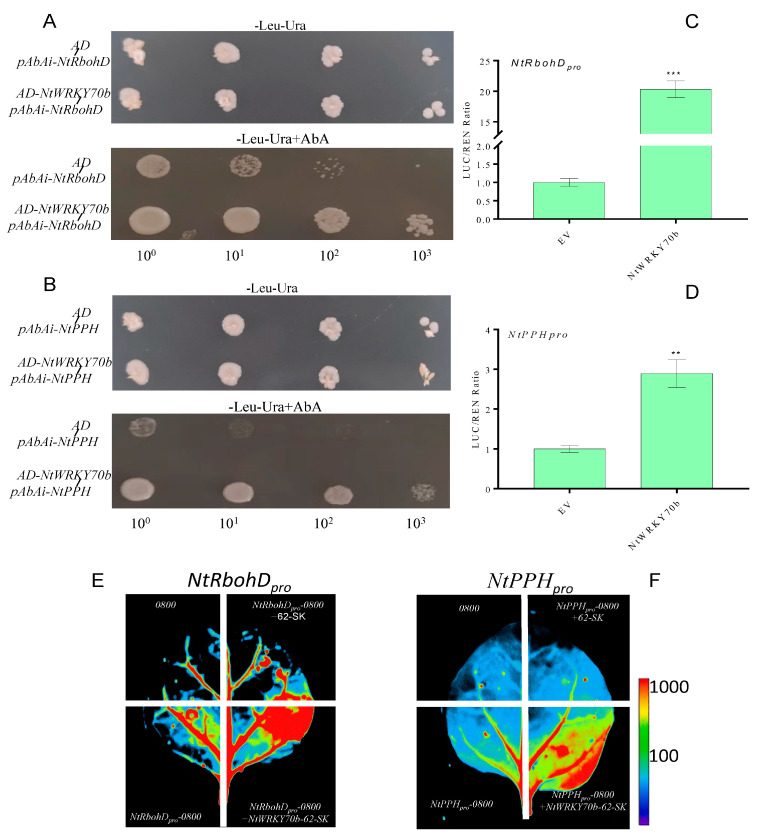
*NtRbohD* and *NtPPH* are the direct target genes of NtWRKY70b. (**A**,**B**) NtWRKY70b binds to the promoters of *NtRbohD* and *NtPPH* in yeast cells. (**C**–**F**) Dual-luciferase reporter assays show that NtWRKY70b directly affects the expression of *NtRbohD* (**C**,**E**) and *NtPPH* (**D**,**F**) in tobacco leaves. Three independent experiments were performed, showing similar results. The data are means ± SD of three biological replicates. Asterisks indicate statistically significant differences levels (Student’s *t* test, ** *p* < 0.01, and *** *p* < 0.001) with corresponding controls.

The observations mentioned above revealed that the expression levels of ROS-synthesis/scavenging-related genes are significantly changed in *NtWRKY70b-OE* lines, and the ROS content is the highest in the overexpression lines. To verify whether NtWRKY70b promotes endogenous ROS accumulation by directly regulating the expression of these genes, we first analyzed the promoter sequences of related genes. We found that the promoter sequences of *NtRbohD* contains one W-box element, which is the conserved sequence bound by WRKY TFs. Furthermore, in the promoter sequences of *NtPPH*, there were several W-box elements (Supplementary Figure S2). These findings indicate that *NtRbohD* and *NtPPH* could be direct target genes of NtWRKY70b. We used yeast one-hybrid analysis (Y1H) to verify whether NtWRKY70b could directly bind to the promoters of the above two genes. The pGADT7-NtWRKY70b construct was co-transfected with *pAbAi-NtRbohD* pro and *pAbAi-NtPPH* pro plasmids into yeast strain Y1HGold, respectively. The experimental results demonstrated that NtWRKY70b directly bound to the promoter sequences of *NtRbohD* and *NtPPH* in yeast cells (Figure 8A,B). Furthermore, we performed the dual-luciferase assay with the *N. benthamiana* leaf system to confirm NtWRKY70b directly binding to the promoter sequences of *NtRbohD* and *NtPPH*. The promoters of *NtRbohD* and *NtPPH* were inserted into the *pGreenII0800* vector, then these recombinant plasmids were transformed into leaves of the *N. benthamiana* through the agrobacterium-mediated method. The observations from the dual-luciferase reporter system demonstrated that NtWRKY70b directly regulates *pGreenII0800-LUC-NtRbohDpro* and *pGreenII0800-LUC-NtPPHpro* in vivo (Figure 8C–F). Overall, these results reveal that NtWRKY70b directly binds to the promoter sequences of *NtRbohD and NtPPH* and regulates their expression, thereby promoting age-dependent leaf senescence.

### 2.8. H_2_S Suppresses Early Aging Phenotype of NtWRKY70b-Overexpressing Plants

**Figure 9:**
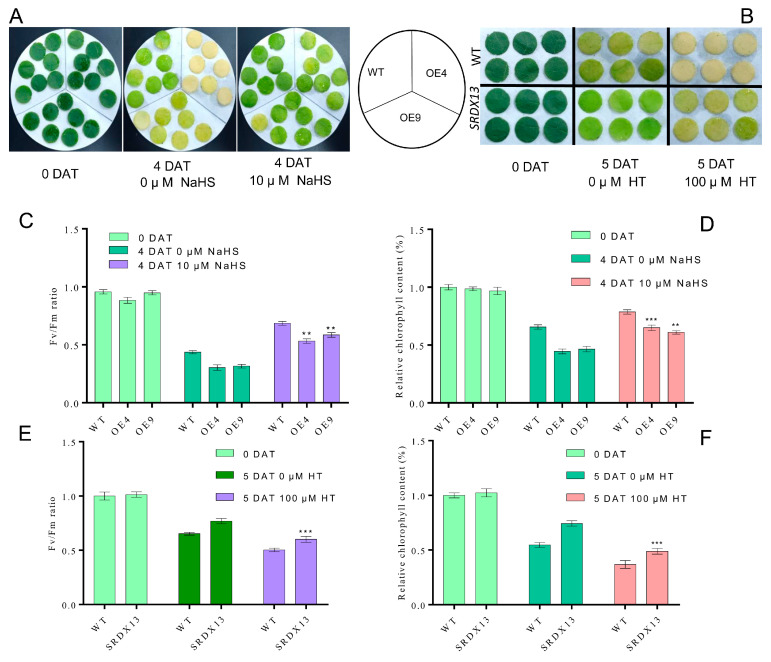
H_2_S represses the premature leaf senescence of *NtWRKY70b-OE* plants. (**A**) Phenotypic analysis of *NtWRKY70b-OE* and WT leaves after four days of 0 and 10 μM NaHS treatment under dark conditions. (**B**) Phenotypic observation of *NtWRKY70b-SRDX* and WT after five days of 0 and 100 μM HT treatment under dark condition. (**C**) Fv/Fm ratio of leaves in (**A**,**D**) Relative chlorophyll content of leaves in (**A**,**E**) Fv/Fm ratio of leaves in (**B**,**F**) Relative chlorophyll content of leaves in (**B**). The data are means ± SD of three biological replicates. Asterisks indicate statistically significant differences levels (Student’s *t* test, ** *p* < 0.01, and *** *p* < 0.001) with corresponding controls.

Emerging studies have revealed that H_2_S plays essential roles in the inhibition of leaf senescence [46,48]. Given our findings that, during leaf senescence, NtWRKY70b suppresses the expression of *NtDCD1* and *NtCYSC1*, two putative H_2_S biosynthesis genes, thereby inhibiting H_2_S production, in order to confirm H_2_S acting as a downstream of NtWRKY70b, we studied whether the modification of H_2_S content in NtWRKY70b-OE lines could influence the accelerated leaf senescence phenotype. To this end, pharmacological tests were performed to check H_2_S functions in the leaf senescence process using sodium hydrosulfide (NaHS, a H_2_S donor) and hypotaurine (HT, a H_2_S scavenger). Upon application of NaHS treatment, these transgenic lines and WT displayed a delayed senescence phenotype compared to CK (Figure 9A), while HT treatment accelerated the progress of leaf senescence (Figure 9B). We also determined the chlorophyll content and Fv/Fm ratio of these NtWRKY70b-OE, NtWRKY70b-SRDX, and WT plants under NaHS and HT treatments (Figure 9C–F). These findings showed that pretreatment with NaHS increased chlorophyll content and decreased electrolytic leakage in plants compared to CK, yet HT treatment triggered opposite results. Collectively, these results indicate that the decreased H_2_S content in *NtWRKY70b-OE* lines serves as a key mechanism underlying its accelerated leaf senescence phenotype.

## 3. Discussion

Senescence is a tightly programmed process of plant cell death, regulated by integrating external environmental and internal developmental signals. Plants have evolved a myriad of highly complex strategies to adapt to changing environmental conditions, such as leaf senescence, thereby enhancing plant fitness and reproduction [50]. An increasing number of studies have revealed that premature senescence significantly affects crop yield. Some stay-green mutants of crop plants could significantly increase biomass and yield. Therefore, dissecting the molecular mechanisms of leaf senescence can provide a theoretical basis for breeding varieties with higher yields [51,52]. This study aimed to revealed the molecular mechanism of NtWRKY70b regulating leaf senescence in tobacco.

Transcription factors, acting as hubs, participate in the leaf senescence process by regulating genes involved in chlorophyll degradation metabolism, aging-related genes, hormone synthesis genes, etc. They bind to specific cis-acting elements in the target gene promoters to regulate downstream gene expression, leading to the activation or inhibition of target genes during senescence. In Arabidopsis, the genes of 96 transcription factors are upregulated at least threefold in senescent leaves [53]. These transcription factors belong to 20 different families, with the largest families being NAC, WRKY, C2H2-type zinc finger, AP2/EREBP, and MYB proteins. WRKY TFs, as one of the largest transcription factor families in plants, play essential roles in the leaf senescence process. In the past decades, transcriptome analysis has demonstrated that a large number of genes are senescence-associated genes (SAGs), but only a small fraction of genes have been functionally characterized. In this study, we cloned and identified a tobacco WRKY TF, designated NtWRKY70b, acting as a positive regulator of natural leaf senescence. The expression profile analysis showed that NtWRKY70b transcript levels were induced by aging and hydrogen peroxide (H_2_O_2_) and downregulated upon hydrogen sulfide (H_2_S) treatment. Overexpression of *NtWRKY70b* (OE) clearly promoted leaf senescence, triggering increased levels of reactive oxygen species (ROS) and decreased H_2_S content, while functional inhibition of NtWRKY70b delays leaf senescence. In summary, our study provides new insights into the connection between transcription factors and leaf senescence.

Previous studies have demonstrated that reactive oxygen species (ROS) serve as important signaling molecules, playing crucial roles in various growth and developmental processes, including plant senescence [35,54]. When ROS accumulate excessively in plant cells, lipid peroxidation is initiated, leading to cell membrane damage and, in severe cases, cell death. As signaling molecules, ROS can regulate plant senescence through complex signaling systems. However, the upstream regulatory mechanism of endogenous ROS biosynthesis in the leaf senescence process is largely unknown. Here, we found that the accumulations of superoxide anions (O_2_^−^), ion leakage, and MDA content in *NtWRKY70b-OE* lines were higher than that in WT, and ROS-scavenging enzyme activity in *NtWRKY70b-OE* was lower. Yet the results of *SRDX13* showed opposite trends. These results indicate that overexpression of NtWRKY70b leads to massive ROS accumulation in senescent leaves. We further explored its molecular mechanisms. In this study, we found that the ROS-synthesis-related genes *NtRbohD* and *NtRbohE* were upregulated in *NtWRKY70b-OE*, while the expression of H_2_S-synthesis-related genes (e.g., *NtDCD1* and *NtCYSC1*) was downregulated. It is well known that hydrogen sulfide (H_2_S) is an important gasotransmitter that could enhance the expression of various stress-resistant genes and signal transduction, alleviating oxidative stress damage to plants [55]. Studies have reported that the blocking function in LCD1 reduces H_2_S production, leading to premature senescence of tomato leaves [46]. In addition, exogenous H_2_S reduces the production of superoxide anions (O_2_^.−^), malondialdehyde (MDA), and H_2_O_2_ in tomato fruits [56]. In summary, H_2_S could negatively regulate tomato leaf senescence, but its regulatory network of regulating leaf senescence in plants remains unclear. In this study, we designed pharmacological experiments involving exogenous application of NaHS and HT. The results indicate that HT can alleviate the stay-green phenotype of NtWRKY70b-SRDX, while exogenous NaHS delays the premature senescence of NtWRKY70b-OE, implying that H_2_S is a key downstream biomolecule of NtWRKY70b. In *Arabidopsis*, 10 Rboh genes may function in a tissue- or organ-specific manner [19]. We found that the expression levels of two Rboh putative homologous genes (e.g., *NtRbohD* and *NtRbohE*) in tobacco were significantly upregulated during leaf senescence, and NtWRKY70b could bind to their promoter sequences and activate their expressions. It is further elucidated that the molecular mechanism of NtWRKY70b accelerates leaf senescence by regulating the accumulation of ROS. From the above results, it can be found that overexpression of NtWRKY70b can change the expression levels of ROS- and H_2_S-related genes, and further studies showed that NtWRKY70b can promote the production of reactive oxygen species by directly binding to the promoters of *NtRbohD* and *NtPPH*, but it has not been found that NtWRKY70b can directly regulate the expression of other genes (e.g., *NtRbohE, NtSOD, NtAPX, NtPOD, NtDCD,* or *NtCYSC1*). So, we can speculate that in addition to NtWRKY70b directly regulating NtRbohD and NtPPH expression, there may be other indirect regulatory pathways and feedback inhibition pathways in the process of NtWRKY70b regulating leaf senescence.

Leaf senescence represents the final stage of leaf growth and development, and its complex regulatory networks have been extensively studied and reported by many researchers. An increasing number of reports have revealed that transcription factors (TFs) act as crucial regulators in regulating leaf senescence. In this study, we identified a novel transcription factor, NtWRKY70b, which played a positive regulatory role in leaf senescence process. Furthermore, these findings indicate that NtWRKY70b directly binds to the promoters of the ROS-synthesis-related gene *NtRbohD* and the chlorophyll degradation gene *NtPPH*, thereby activating their expression and subsequently promoting ROS accumulation and accelerating cellular oxidation and chlorophyll degradation processes, ultimately leading to premature leaf senescence. Intriguingly, we found that the overexpression of NtWRKY70b caused decreased expression levels of the H_2_S synthesis genes (e.g., *NtDCD1* and *NtCYSC1*), inhibiting endogenous H_2_S synthesis then accelerating leaf senescence. It will be important to study the molecular mechanism of H_2_S participates in the regulation of leaf senescence. In Arabidopsis, it was reported that AtWRKY70 TF played a negative regulatory role in leaf senescence [24]. The difference with our observations may be due to the functional specificity of WRKY TFs derived from different plant species. So, it will be interesting to further investigate the transcriptional and translational regulatory network of NtWRKY70b taking part in regulating the onset and progress of plant senescence, and whether this mechanism is conserved for other plant species. A deeper understanding of the molecular and physiological mechanisms of H_2_S-mediated leaf senescence is crucial for advancing agricultural practices, optimizing crop productivity, and managing natural ecosystems in a changing environment.

## 4. Materials and Methods

### 4.1. Plant Materials and Growth Conditions

All transgenic plants used in this study were in the *Nicotiana tabacum* L. Cv. “K326” background. *Nicotiana benthamiana* was used for transient transformation assays. Those seeds mentioned above were surface-sterilized with 75% ethanol and grown on 1/2 Murashige and Skoog medium containing 20 g/L sucrose, 0.5 g/L MES and 1.1% agar (PH = 5.8) for 10 days. The seedings of those lines were transferred to soil to continue growth under the same growth conditions (28 °C, 16 h light/8 h dark and 55% relative humidity). When cultivating “K326” to 12 true leaves, the 3rd, 6th and 10th leaves from the top were named as young leaves (YL), mature leaves (ML), and senescence leaves (SL), respectively. The 5-week-old *Nicotiana benthamiana* leaves were selected for subcellular localization and transient transformation experiments.

### 4.2. RNA Extraction and Quantitative Real-Time PCR Analysis

Total RNA was extracted from “K326” using the M5 Total RNA Extraction Reagent (Mei5bio, Beijing, China) according to the manufacturer’s instructions, and first-strand cDNAs were synthesized by the HiScript III RT SuperMix by qPCR (Vazyme, Nanjing, China). qRT-PCR was performed on Thermal Cycler Dice^®^ Real-Time System III (TaKaRa, Kusatsu, Japan) by using SYBR Green Master Mix as described previously [4]. The *NtActin* was used as an internal control gene, and the expression levels of genes were calculated with the 2^−ΔΔCT^ method. Primers used for qRT-PCR are listed in the Supplementary Table S2.

### 4.3. Subcellular Localization of NtWRKY70b Protein

To clarify the subcellular localization of the NtWRKY70b protein, the CDS sequence of the *NtWRKY70b* gene was connected with linearized *2301-S2-GFP* vector to construct *2301-S2-GFP-NtWRKY70b* fusion expression vector, which was transformed into the Agrobacterium strain GV3101. *2301-S2-GFP-NtWRKY70b* and the nucleus marker were injected into the leaves of 1-month-old *Nicotiana benthamiana*. After 1 d dark treatment and 2 d long-day condition (16 h light/8 h dark), the fluorescence signals were observed by laser confocal microscope (LEICA TCS SP5II, Wetzlar, Germany).

### 4.4. Transactivation Activity Assay

To explore transactivation activities of NtWYKY70b protein, the CDS fragment of *NtWYKY70b* was amplified by PCR and fused in-frame to the BD domain of pGBKT7 vector, yielding the pGBKT7-NtWYKY70b fusion constructs. The recombinant plasmids of pGBKT7-NtWYKY70b, pGBKT7-AD, and the empty vector pGBKT7 were transformed into yeast strain AH109, respectively. Yeast transformants harboring the above plasmids were plated on SD/-Trp and SD/-Trp/-His medium and incubated at 28 °C for 72 h. We attached the colony to the filter paper and then transferred the filter paper to liquid nitrogen and froze it for 20 s. We removed the filter paper and melted it at room temperature. We repeated the freezing and thawing process three times. Transactivation activity of the fused NtWYKY70b protein was assessed by the growth and production of blue pigments.

### 4.5. Generation of Transgenic Plants

For *NtWYKY70b* overexpression lines, the CDS fragment of *NtWYKY70b* was cloned into *pCAMBIA2301-S2* vector under the control of cauliflower mosaic virus (CaMV) 35S promoter, constructing the *pCAMBIA2301-S2-NtWYKY70b* recombinant plasmid. For *NtWYKY70b -SRDX* lines, the coding region sequence of *NtWYKY70b* without termination codons was used as a template, and an SRDX inhibitory domain was added at the end of the reverse primer to construct *NtWYKY70b-SRDX* expression vector. Those above fused plasmids were transformed into Agrobacterium strain EHA105, then introduced into leaves of “K326” through Agrobacterium-mediated transformation. The transgenic lines were screened through planting seeds on MS medium containing 200 mg/L kanamycin.

### 4.6. Transient Transformation of NtWYKY70b

To study the transient expression, the coding sequence of *NtWYKY70b* was amplified and a w*pCAMBIA2301-S2* vector was inserted, yielding the *pCAMBIA2301-S2-NtWRKY70b* construct. *pCAMBIA2301-S2-NtWRKY70b* recombinant plasmid and *pCAMBIA2301-S2* empty plasmid (CK) were transformed into Agrobacterium strain GV3101, respectively. Those above strains were re-suspended in solution for injection (10 mmol/L MES, 200 mmol/L AS, 10 mmol/L MgCl_2_, pH = 5.7), then infiltrated into leaves of 1-month-old *Nicotiana benthamiana* to explore the transient expression of NtWRKY70b. After dark treatment for 24 h and normal condition for 48 h, the infected leaves were detached for dark-induced assay. Under dark conditions for another 5 d, the phenotypes were observed as described previously [4].

### 4.7. Leaf Senescence Assay

For phenotypic assay, seeds of WT, NtWRKY70b overexpression, and NtWRKY70b-SRDX lines were surface-sterilized with 75% ethanol and grown on 1/2 MS medium [2.2 g/L MS, 0.5 g/L MES, 20 g/L sucrose, 200 mg/L Kanamycin and 1.1% agar (PH = 5.8)] for 14 d, then transferred to soil to continue growing under the long-day condition at the same time. The leaves from any genotype plants were detached and defined as (1), (2), and (3), respectively. Leaves of 2-month-old different genotypes were harvested for phenotype observation and measurement of physiological values.

### 4.8. Physiological and Biochemical Assays

Two-month-old leaves of WT and different transgenic lines were detached for physiological and biochemical measurements.

The chlorophyll content was measured as described previously [6]. Briefly, chlorophyll contents were measured by the colorimetric method, and three biological replicates were performed. The leaves of different genotype plants were cut into pieces and flash-frozen using liquid nitrogen. Chlorophyll was extracted from leaf samples with 95% ethanol, and then chlorophyll content was counted according to the absorbance at 649 nm and 665 nm. The Fv/Fm was measured by a Hansatech m-pea fluorescence spectrometer.

For ions leakage rates, leaves were placed in deionized water and placed in a vacuum pump for 30 min, after which the conductivity was measured as E1. Then, the leaves were boiled for 10 min and cooled down to room temperature, and the conductivity was measured as E2. Ions leakage rates (%) = E1/E2 × 100.

The malondialdehyde (MDA) accumulation was measured by the thiobarbituric acid-based method. In brief, the leaves were cut into small pieces, then mixed with 10% TCA and 0.6% TBA. After being boiled for 15 min, the leaves were cooled down to room temperature, and absorbance was measured at 532 nm, 600 nm, and 450 nm by using a spectrophotometer. The MDA content was measured as described [39].

The superoxide anions (O_2_^−^) content was measured by superoxide anion assay kit (Nanjing Jiancheng). The superoxide dismutase (SOD) and catalase (CAT) activities were analyzed by SOD Detection Kit (Nanjing Jiancheng) and CAT Detection Kit (Nanjing Jiancheng) according to the operating instructions. The enzyme liquid was extracted for determination of superoxide dismutase (SOD) as well as catalase (CAT) activities according to a previous study [39]. Three replicates were performed in these experiments.

The hydrogen sulfide content was measured as described previously [27].

### 4.9. Yeast One-Hybrid Analysis

The CDS fragment of *NtWYKY70b* was cloned and fused to *pGADT7* vector, and the *NtRbohD* (880 bp upstream from ATG) and *NtPPH* (680 bp upstream from ATG) promoter sequences containing the NtWYKY70b-binding sites were inserted into a pAbAi vector. Those above vectors and empty vector were inserted into yeast strain Y1HGold. The control and experimental groups were grown on SD/-Ura-Leu and SD/-Ura-Leu + AbA (50 ng/mL and 100 ng/mL) medium to evaluate the DNA binding activity of NtWYKY70b by observing the growth status of the yeast cells at 28 °C for 3 d.

### 4.10. Dual-Luciferase Assay

The promoter sequences of *NtRbohD* and *NtPPH* were amplified from “K326” cDNA by PCR, respectively, and then inserted into pGreenII0800-LUC vector as a reporter plasmid. The CDS fragment of *NtWYKY70b* was fused with the vector pGreenII 62-SK as an effector plasmid [27]. Those above plasmids were infiltrated into leaves of 1-month-old *Nicotiana benthamiana* through Agrobacterium-mediated transformation. The ratio of LUC/REN was detected by Dual-Luciferase Reporter Assay System Kit (Promega, Madison, WI, USA) following the manufacturer’s instructions.

## Data Availability

The data that support the findings of this study are available from the corresponding author upon reasonable request.

## Supplementary Materials

The supporting information can be downloaded at: https://www.mdpi.com/article/10.3390/ijms25073686/s1.
