# Supplementary material for: Tobacco Transcription Factor NtWRKY70b Facilitates Leaf Senescence via Inducing ROS Accumulation and Impairing Hydrogen Sulfide Biosynthesis
Source: Int J Mol Sci. 2024 Mar 26;25(7):3686. doi: 10.3390/ijms25073686 (PMC11012213; doi:10.3390/ijms25073686)
Supplement: Supplementary file 1 [file ijms-25-03686-s001.zip › Supplementary Figure S1.pdf]

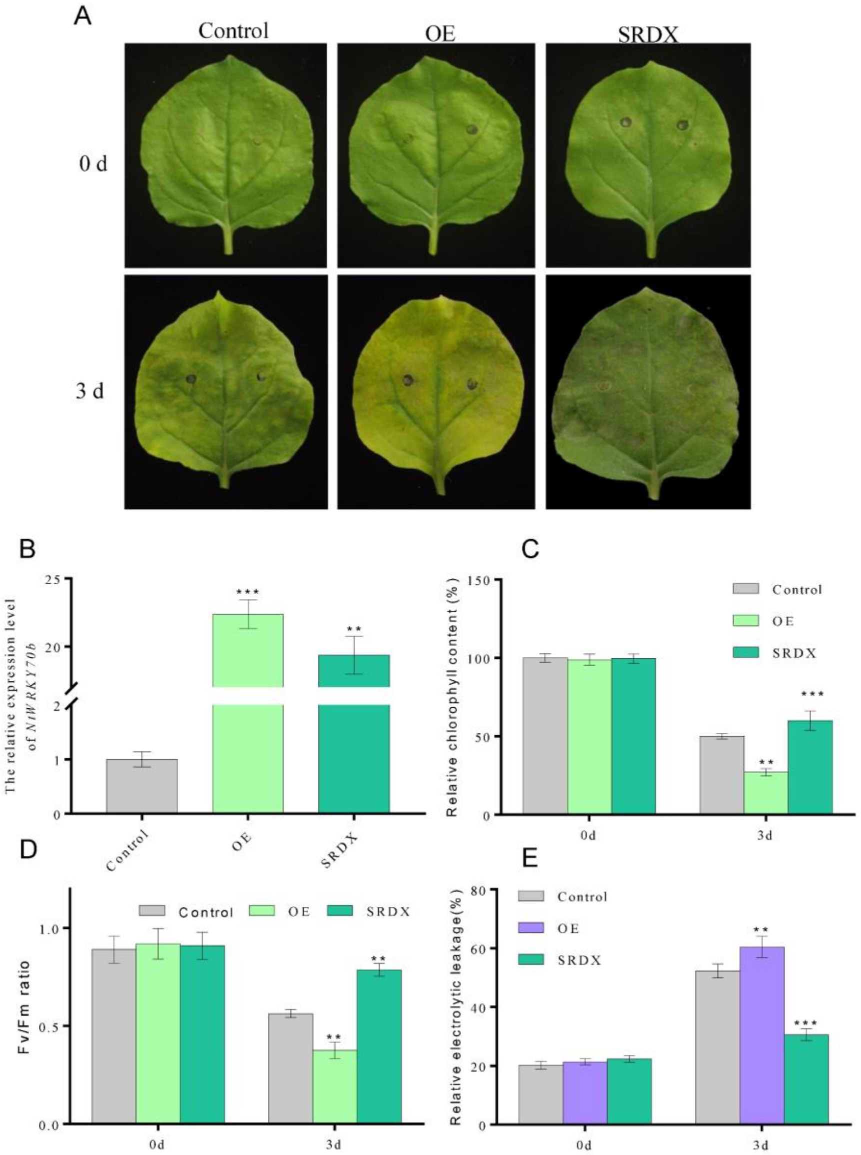

Supplementary Figure S1. Phenotypic analysis of transiently transformed leaves under dark conditions. (A) Phenotype observation of these transiently transformed leaves under darkness for 3 days. (B) The expression level of NtWRKY70b in transiently transformed tobacco leaves. (C) chlorophyll content (D) Fv/Fm (E) ion leakage rate. Three biological repeats were performed. Error bars indicate standard deviation (n=10). Asterisks indicate significant differences (Student's t test,**p < 0.01, ***p <0.001) with corresponding controls
