# Supplementary material for: Tobacco Transcription Factor NtWRKY70b Facilitates Leaf Senescence via Inducing ROS Accumulation and Impairing Hydrogen Sulfide Biosynthesis
Source: Int J Mol Sci. 2024 Mar 26;25(7):3686. doi: 10.3390/ijms25073686 (PMC11012213; doi:10.3390/ijms25073686)
Supplement: Supplementary file 1 [file ijms-25-03686-s001.zip › Supplementary Figure S2.pdf]

NtRbohD promoter sequence

GTAGTTGTCTACGTCGTTGCGGCAACTGTGGAGTATAAATTATTGAGATAACATTAGTCTTAAAATAAATTTATTTGAAGAAAA

ATAAGAATAAGGGCGTGTAATAACAACGGCATGGGCATGCATGTATCGGTAAAATAATGCATATGACATAAACAAAAATATTC

TTTGGTCAAACAAAGACCTACCCCCTTCATTTCCTAGCTCTCGCCCTTTTTCTTTTTTCTTTTTCAACAGAATGCGGCTTCTTC

CCACCATTATATAACTAAAGTTGAAGGCCGAAGGAACGACCACTTCTGTATTCTGCTTGGAACAAGAAAAATCCCCAATCTT

TTATTTGTTTATTAAAATTAGTACGCCAAGAAAGAAAGAAAGAAAGACAGAAAGACTCGGTCTTCTTTCTTCTCTTGGTCTG

AAACTCCAAAATAGAATACCAATTATTAATCTTTTGTCATCTTTTTCCTTCT

NtPPH promoter sequence

GAGTTATGGCAGTAAATTCATACGGTTGGCTAAACAGAACTATGCGATAAGATATAGCAATATTCGTGAATTCAACA

AAGTACCCAGCGAACAACTTCAGTACACTGATAAATGCCCAAAGGAACATCTTTATCAAGATCTACATATGAAACCT

AGATATTGGGCACACTTACAAGGTCAAAATCGTACAGGATGCATAATGGAAGGTAGAAACAATTACGAGATTGGAA

GGATTCCGACTACAGGTTGTGGTGTGATAAGGAAGCCTAAGGGGGGAGTGACCTGAACTTTGGATTTATTCACAGAA

CAACTGCCTAAATGGCAAGGAAAGTATTGAGGTATTCACAAGAGCTATAAGTTATGAAAATGATAAGGGCATCAGT

CAACATTCGAGGACGAATGTTCCAAAAGGGGGAATAATGTTACGCCCCGCAATATTACATCGATATCACGTTTCACA

ATATTAAGTTACGACAATGTTGCACCCTGAAGTATTAAACGTGGAATTTGTCGTAAGGTAATTGACATCAGTCCAAG

TAAAAGATTATTTGGAGGTTATAAGGATTATGCTATTTCACAAGTGATTAGTAAATTCGTGAAGGTGAGAAGGGAAG

CAAGTCGAAGAAAATGAATTTCGTTAAAATATTTAAGAGACTAAATATGCTCAGGGTTATATTTGAGGGACCAAAAT

AGACATACCCTCAAAGATAAGAGACAATATTGGCCAAAAACTCTTCAAGAGCTCATGATAATTTTCAAATTTACAAA

ACCTTTCTATTAAAATATTAATGTAACAAGTATTTTTCAATAATTCGAACTACACATTATAATACTTTATAGTTCATAT

CGACTAAATTTGACCATTCTGTTTACTTATACGTAAACTAATTAATGTAAGGGAAGTAAAATTCTTTATGTAAAAATA

AGAACATCCTTATATAACAATGCTTCACTATAAAAGTTAAATTTTTTCGGAACCACATATTATGTTTTGTTATAATAT

ATGTTCTCTATAATAACAATTCA

Supplementary Figure S2. W-box in these promoter of *NtRbohD and NtPPH*. W-box: the red font highlighted in the background.
