# Supplementary material for: Tobacco Transcription Factor NtWRKY70b Facilitates Leaf Senescence via Inducing ROS Accumulation and Impairing Hydrogen Sulfide Biosynthesis
Source: Int J Mol Sci. 2024 Mar 26;25(7):3686. doi: 10.3390/ijms25073686 (PMC11012213; doi:10.3390/ijms25073686)
Supplement: Supplementary file 1 [file ijms-25-03686-s001.zip › Supplementary tables-WRKY.pdf]

Supplementary Table S1. The GenBank accession numbers of NtWRKY70b and its close homologues proteins.

Name	Accession	Name	Accession
AtWRKY70	AAL13046.1	NtWKY70b	XP_016436463.1
StWRKY70	XP_015158414.1	VrWRKY70	KAJ9694195.1
MnWRKY70	XP_010098011.1	HuWRKY70	XP_021290890.1
CsWRKY70	KAH9673391.1	PtWRKY70	KAG6769652.1
CsWRKY70	KAF5941069.1	LtWRKY70	GLL27008.1
MeWRKY70	XP_021622706.1	HbWRKY70	XP_021664309.2
PtWRKY70	XP_002323675.2	InWRKY70	XP_019186254.1
CaWRKY70	XP_016543153.1	EgCAT1	XP_010051967.2

Supplementary Table S2. The qRT-PCR Primers sequences used in this study.

Name	Forward primers (5’-3’)	Reverse primers (5’-3’)
NtWRKY70b	5′-ATCCTCTAGAGTCGAATGAAGAAGCCAT TAGTTCATGA-3′	5′-ATGCCTGCAGGTCGATCAAGAAAACTCTAT GGCCTCATC-3′
NtActin	5′-ACCTCTATGGCAACATTGTGCTCAG-3′	5′-CTGGGAGCCAAAGCGGTGATT-3′
NtCP1	5′-CAGTGGCTAATCAACCTGTTTCGG-3′	5′-ACACCACTTGAATAGAACTGGAAATCG-3′
NtRBCS	5′-CGAAACTCTCTCATACCTTCCCGA-3′	5′-CATGGTCCAGTATCTGCCGTCATA-3′
NtSOD	5′-CTCCTACCGTCGCCAAAT-3′	5′-GCCCAACCAAGAGAACCC-3′
NtCAT	5′-AGGTACCGCTCATTCACACC-3′	5′-AAGCAAGCTTTTGACCCAGA-3′
NtPPH	5′- AGAATCTTTTAAACTGCCATCAA -3′	5′- AATGAATTTTGTGGCCTCGCCAT-3′
NtRbohD	5′- -CAACAGCCACGTCCTTTTGA -3′	5′- CCCGAGGAAGTAAGCCATCT -3′
NtRbohE	5′- TTCGGAAAATCATCACCCGC -3′	5′- ATCATCATTGGACTTGGCGC -3′
NtDCD1	5′- CAGCTCACCATTTTCTTTGCAAC -3′	5′- AGTCAGCCCCCTGTGCCACAGCAT -3′
NtCYSC1	5′- GCATTTATGGCAGCAATGAAA -3′	5′- CTCAGGACCAGTCGTCTCAA -3′
